# Antifungal Activity of Aromatic Plants of the *Lamiaceae* Family in Bread

**DOI:** 10.3390/foods9111642

**Published:** 2020-11-10

**Authors:** Adriana Skendi, Dimitrios Ν. Katsantonis, Paschalina Chatzopoulou, Maria Irakli, Maria Papageorgiou

**Affiliations:** 1 Hellenic Agricultural Organization—Demeter, Institute of Plant Breeding and Genetic Resources, 57001 Thessaloniki, Greece; dikatsa@cerealinstitute.gr (D.N.K.); chatzopoulou@ipgrb.gr (P.C.); irakli@ipgrb.gr (M.I.); 2 Department of Food Science and Technology, International Hellenic University, 57400 Thessaloniki, Greece; mariapapage@food.teithe.gr

**Keywords:** antifungal activity, *Aspergillus niger*, bread, essential oil, GC-MS, HPLC, Greek oregano, *Penicillium*, phenolics, Satureja, thyme

## Abstract

The antifungal effect of aromatic plants (oregano, thyme, and Satureja) in dry form and as essential oils was evaluated in vitro (in potato dextrose agar (PDA)) and in bread against two phytopathogenic fungi found in food (*Aspergillus* *niger* and *Penicillium*). Gas and liquid chromatography were used to analyze essential oils attained by hydrodistillation of the aerial parts of the aromatic plants and of the dried plant aqueous solutions that were autoclaved for 20 min at 121 °C before analysis. Carvacrol, α-pinene, p-cymene, and γ-terpinene were the main components of the essential oils, whereas carvacrol, rosmarinic and caffeic acids were the main components of the water extracts. In vitro antifungal test results showed that the addition of plants in dry form had great antifungal potential against both fungal strains studied. *Penicillium* was more sensitive to the presence of aromatic plants than *Aspergillus*. Among the three plant species tested, thyme was the most potent antifungal against both fungi. For the bread product, all three aromatic plants studied showed inhibitory effects against both fungi. Results presented here suggest that oregano, thyme and Satureja incorporated in a bread recipe possess antimicrobial properties and are a potential source of antimicrobial ingredients for the food industry.

## 1. Introduction

Generally recognized as safe (GRAS), aromatic plants are excellent alternatives for chemical additives used in the food industry. Plants of the *Lamiaceae* family, mainly found in the Mediterranean region, are utilized to produce essential oils that show antioxidant and antimicrobial properties [1]. Consumer demand for less processed foods, together with the increased concern about the use of synthetic preservatives, have directed the interest of the food industry towards natural sources of preservatives. Essential oils have been used in vitro for their antimicrobial and antioxidant properties in different fungi and bacterial species, aiming to use them as a promising technology in order to increase the shelf life of foods [2,3,4,5].

The essential oils of the *Lamiaceae* family have been extensively studied with respect to their use as food preservatives [1,6,7], and among them, oregano and thyme are of major importance due to their antimicrobial and antifungal activities [6,8,9,10,11,12]. Essential oils are usually extracted from plants by steam distillation, and they contain a variety of volatile molecules such as terpenes and terpenoids, phenol-derived aromatic components, as well as aliphatic compounds [13,14]. Carvacrol, an essential oil compound present in several taxa of the *Lamiaceae* family, is known to act as a natural preservative [15,16]. It was reported that carvacrol interacts with the membrane of bacteria by changing its permeability, resulting in the dissipation of ion gradients that in turn leads to impairment of essential processes in the cell, resulting in cell death. On the other hand, carvacrol does not exhibit adverse effects on human health, since the levels at which it exhibits its antimicrobial activity are not toxic at the cellular level [17].

It was reported that synergism exists between carvacrol and its precursor, p-cymene [16]. Genotype, as well as environmental and agronomic conditions, affect the chemical composition of essential oils with consequences for their antibacterial/antifungal activity [16,18]. Carvacrol is the main phenolic compound of oregano, thyme and Satureja [19].

The majority of the studies in the literature are focused on the evaluation of the antifungal/antibacterial effect of the essential oils, ignoring the real effect of the plant material and the potential efficacy of other groups of compounds. By incorporation of the aromatic plant in their dry form in vitro, we tried to evaluate their antifungal effect for the first time. Moreover, we investigated the antifungal effect of aromatic plants by incorporating them in the bread matrix for the first time.

The objective of the present work was to explore the antifungal activities of three aromatic plants (in the dry form) common to the Mediterranean area, and widely used as spices (*Origanum vulgare* ssp. *hirtum* (oregano), *Thymus capitatus* (thyme) and *Satureja thymbra* (Satureja)) in potato dextrose agar (PDA) against two fungi, *Aspergillus niger* van Tieghem (BPIC 2539) and *Penicillium,* isolated from bread. Moreover, the antifungal activities of the selected plants (when added as essential oil or in dry form) in a bread matrix were also studied, while possible relationships were evaluated between the components of oregano, thyme and Satureja and their antifungal activity. Finally, our breadmaking trials determined, for the first time, the extent of inhibition that these aromatic plants have when applied in bread at concentrations that are acceptable from a sensorial–organoleptic point of view, as established in one our previous studies [20].

## 2. Materials and Methods

### 2.1. Plant Materials and Chemicals

Antifungal activity tests were performed for *Origanum vulgare* ssp. *hirtum* (oregano), *Thymus capitatus* (thyme), and *Satureja thymbra* (Satureja). Samples were provided by the Hellenic Agricultural Organization—Demeter, Plant Breeding and Genetic Resources Institute, (Thessaloniki, Greece). The aerial parts (flowers and leaves) of aromatic plants were collected in summer 2014, dried in the shade at ambient temperature, and ground into powder to pass through a 0.5 mm sieve as reported in [20].

Potato dextrose agar (PDA) was purchased from Merck (Darmstadt, Germany), whereas acetonitrile, methanol, and water (HPLC gradient grade) were supplied by Chem-Lab (Zedelgem, Belgium). Other chemicals and reagents were of HPLC or analytical grade.

The investigated fungi *Aspergillus niger* van Tieghem (BPIC 2539) was a donation from the collection of Benaki Phytopathological Institute (Athens, Greece), whereas *Penicillium* fungi was isolated from bread purchased in a local supermarket, according to the procedure in Section 2.2. below.

### 2.2. Isolation and Identification of Pencillum

Bread slices were left in open air at room temperature to develop green mold. Fungal isolation from the overgrown colonies was carried out by touching the tip of the conidiophores and transferring them into sterilized water to dilute and inoculate PDA petri dishes for monoconidial isolation. Colonies belonging to the *Penicillium* genera were identified according to their macromorphological characteristics, transferred onto PDA, and incubated for 7 days at 25 ± 2 °C [21] in order to obtain pure stock cultures, which were stored at 4 °C. The identification of *Penicillium* genera was conducted based on colony diameter, color, and texture, as well as by observing microscopic characteristics such as hyphae and conidiophore appearance, and the size and shape of vesicles, metulae, phialides, and conidia [22].

### 2.3. Preparation of Essential Oils

Fifty grams of each dried comminute plant material from oregano, thyme and Satureja was submitted, separately, to a hydrodistillation process (450 mL of deionized water) in a Clevenger-type apparatus for 2 h. The essential oil was collected, then dried over anhydrous sodium sulphate (Na_2_SO_4_) and finally stored at 4 °C in the dark for further use.

### 2.4. Gas Chromatography/Mass Spectrometry

The essential oils were analyzed by GC/MS as reported previously [23] in order to detect the potential relationship between chemical composition and biological activity as antifungal agents. The analyses were performed by using a gas chromatograph 17A Ver. 3 (Shimadzu Scientific Instruments, Inc., Kyoto, Japan) interfaced with a mass spectrometer Shimadzu QP-5050A, supported by the GC/MS Solution Ver. 1.21 software. The GC was equipped with a fused silica DB-5 capillary column (30 m × 0.25 mm, film thickness 0.25 μm), and the optimal temperature program used was as follows: (a) 55–120 °C (3 °C/min), 120–200 °C (4 °C/min), 200–220 °C (6 °C/min), and 220 °C for 5 min; (b) from 60 to 240 °C with temperature rate 3 °C/min. The injector temperature was maintained at 260 °C; interface temperature 300 °C, ion source heating 200 °C, carrier gas helium (54.8 kPa); split ratio 1:30. The volume injected was 1 μL. Mass spectra were recorded at 70 eV. The acquisition mass range was *m*/*z* 41–450 at a scan time of 0.5 s.

The identification of the constituents was based on comparisons of the retention time with those of authentic samples, comparing their linear indices relative to a series of n-alkanes (C7–C22). Further identifications were also made using a library of mass spectra built up from pure substances and components of known oils, and MS literature data (National Institute of Standards and Technology (NIST), Gaithersburg, MD, USA) [24]. The percentage of each component identified was determined as the average of two replicates.

### 2.5. HPLC Analysis

Aqueous solutions containing 1% (w/v) dry matter of aromatic plants were prepared in glass bottles and autoclaved for 20 min at 121 °C. Aliquots of each plant extract were filtered through a 0.22 µm nylon filter before injected to the HPLC system. HPLC analysis was performed utilizing an Agilent 1200 system (Agilent Technology, Urdorf, Switzerland) equipped with a quaternary pump and a rheodyne injector valve with a 20 µL loop.

Phenolic compounds in the water extract were separated in a Nucleosil 100 C18 5 µm (250 mm × 4.6 mm) column maintained at 30 °C as explained in detail elsewhere [19]. The extracts were analyzed for the presence of 24 phenolics at least in duplicate and the results were expressed as micrograms per g (µg/g). The phenolic compounds were detected utilizing a diode-array detector (DAD) at different wavelengths as follows: 260 nm for protocatechuic acid (PRCA), 4-hydroxybenzoic acid (4-HBA), vanillic acid (VA) and rutin (RUT); 270 nm for gallic acid (GA), (−)-epigallocatechine (EPIG) and syringic acid (SRA); 280 nm for (+)-catechin (CAT), (−)-epicatechin (EPI), trans-cinnamic acid (CIN), (±)-naringenin (NAR), carvacrol (CAR) and thymol (THY); 320 nm for caffeic acid (CA), p-coumaric acid (PCA), ferulic acid (FA) and sinapic acid (SA); 330 nm for chlorogenic acid (CLA), rosmarinic acid (RMA) and kaempferol (KAM); and 360 nm for myricetin (MYR), quercetin (QUE), luteolin (LUT) and apigenin (API).

Chromatograms were recorded and integrated using Agilent Chemstation software (version B.04.01, Agilent Technologies, Santa Clara, CA, USA).

### 2.6. Bread Preparation

The recipe and breadmaking procedure is described in detail elsewhere [20]. The recipe for bread production consisted of wheat flour (300 g), salt (6 g), dry yeast (5 g), water (49.3% on flour basis), and dried aromatic plants at four different levels (0%, 0.25%, 0.5% and 1% in 100 g flour basis) or essential oil at four addition levels (0, 12.5, 25, and 50 μL). All ingredients were mixed for 5 min in a farinograph bowl and then a two-step bulk fermentation (30–32 °C) and proofing up to the optimum volume was performed. Bread loaves were baked for at least 23 min at 210 °C then cooled for at least one hour at room temperature, sealed in polyethylene bags, and stored in the freezer. Before the test, bread loaves were allowed to reach room temperature and were then immediately sliced and sized, and finally placed in petri dishes. The level of aromatic plants and essential oil added in the present study was determined by the results of sensory tests performed and described elsewhere [20].

### 2.7. Preparation of Media and Fungal Spore Suspension

The effect of the addition of 1% dry aromatic plants on *Aspergillus niger* and *Penicillium* growth was determined on petri plates with PDA media. PDA was prepared in 400 mL aliquots. The amount of dry aromatic plants to obtain concentrations of 0.5% and 1% (w/v) were added to PDA before water addition, and then the suspension was autoclaved at 121 °C for 20 min. After cooling to 45 °C, the solutions containing PDA alone or with the added dry aromatic plants were poured into sterile petri plates. Care was taken to ensure that the suspended particles of aromatic plants were evenly distributed within the petri plates.

The fungal conidia that were sub-cultured for 7 days on plates with PDA for mass production were collected by scraping the conidial layers formed on the plate surface using a sterilized scalpel [21,25]. Preparation of the fungal spore suspension was made with spores that were harvested with a sterile loop in sterile distilled water and aseptically transferred into sterile test tubes [21]. The spore suspensions were adjusted to give a final spore concentration of 15 × 10^6^ spores/mL using a hemocytometer.

### 2.8. Inoculation and Incubation of PDA Petri Dishes

Petri plates were centrally inoculated by spotting 10 µL of a spore suspension (1.5 × 10^3^ spores/mL) in the middle of the plate (one inoculum per plate). The control sample was inoculated by spotting 10 µL of sterile distilled water. The plates were sealed with parafilm and then incubated at 25 °C for 12 days. Antifungal activity was evaluated by calculating the area of mycelium growth [12]. The determination was performed for at least 12 biological replicates (petri dishes). A high-resolution photo of each petri dish was analyzed utilizing the eCognition Developer 9 software from Trimble (Trimble Germany GmbH, Munich, Germany).

### 2.9. Inoculation and Incubation of Bread Samples

Bread samples, 8 cm in diameter and 12.5 mm in thickness (whole slice) in Petri dishes, were exposed to ultraviolet light for 30 min and then inoculated with 10 µL of the suspension containing 15 × 10^6^ spores/mL. The bread inoculated with fungal spores were sealed with parafilm and then incubated at 25 °C for 10 days. The determination was done for at least 12 biological replicates (bread slices in petri dishes). A high-resolution photo of each bread slice was analyzed similarly to Section 2.8.

### 2.10. Statistical Analysis

The determination of the effect of aromatic plants on the growth of *Aspergillus* and *Penicillium* species was carried out in two replications. One-way analysis of variance (ANOVA) followed by Duncan’s multiple range test was used to determine significant differences between the means of colony surface of PDA/bread samples on the same day of storage. A general linear model was used to detect the general effect of the type of aromatic plant, level of addition, storage day and fungi species. Significance for all tests was determined at a *p*-value of ≤0.05, with statistical analyses performed using IBM SPSS Statistics for Windows (Version 25.0, IBM Corp. Armonk, NY, USA).

## 3. Results and Discussion

### 3.1. Composition of Essential Oil

The content (% of essential oil) of the compounds determined in the essential oils of the three aromatic plants under investigation varied depending on the plant species (Table 1). There were 19, 20 and 25 compounds identified that represent 99.1%, 99.4%, and 99.8% of the essential oils of oregano, thyme and Satureja, respectively.

Table 1 reveals that the essential oil of oregano consisted mainly of monoterpene phenolic compounds (82.64%), monoterpene hydrocarbons (12.61%) and fewer sesquiterpenes (2.14%) and other oxygenated monoterpenes (1.12%). The main component is carvacrol (82.48%), followed by p-cymene (5.00%), and γ-terpinene (2.62%). Other major components are β-mycene (1.61%), followed by trans-caryophyllene (1.23%), α-terpinene (0.97%) and β-bisabolene (0.91%). The amount of carvacrol in our samples is significantly higher (82.48% vs. 59.7%) than those that Lagouri et al. [26] found in a sample of Greek oregano, whereas the amount of thymol was significantly lower (0.16% vs. 13.70%). The species *Origanum vulgare* ssp. *hirtum*, also known as Greek oregano, is widely distributed throughout the Mediterranean basin [27]. Analysis of the composition of its essential oil, as reported in the literature, showed that the main components are carvacrol accompanied by two monoterpenic hydrocarbons, p-cymene and γ-terpine. Carvacrol and thymol participate in giving the plant its aroma. Generally, the amounts of carvacrol and thymol are negatively correlated. The same applies between their sum and the sum of the two monoterpenic hydrocarbons [28,29].

The main chemical group identified in *Thymus capitatus* essential oil was that of monoterpene phenolic compounds (99.37%), with the main representative being carvacrol (76.19%), followed by p-cymene (7.18%) and γ-terpinene (4.9%). It is known that γ-terpinene and the p-cymene are precursors of thymol and its isomeric carvacrol [30,31].

The chemical composition of the essential oil of *Satureja thymbra* is shown in Table 1. The main chemical group for Satureja essential oil was monoterpene phenolic compounds (46.33%), with carvacrol (44.93%) accounting for approximately 97% of this group. Monoterpene hydrocarbons accounted for 44.99% of the total essential oil compounds present, with γ-terpinene being the main representative compound (29.28%).

Carvacrol and thymol are two components that are present not only in the essential oil obtained from these aromatic plants, but also in aqueous methanolic extracts. In one of our previous studies, HPLC analysis showed that the content of phenolic compounds in oregano, thyme and Satureja was 49.99, 41.07, and 39.93 mg/g, respectively [19], with carvacrol being the main phenolic compound present in the dry aromatic plants.

### 3.2. Antifungal Activity of Aromatic Plants in Petri Dishes

Since the direct addition of aromatic plants in dry form in PDA media is impossible due to the inherent microbial load that the dry material carries, the dry form was mixed with the PDA before sterilization and subsequently sterilized at 121 °C for 20 min. Thus, the amount of phenolic compounds extracted from the dry mater in the aqueous solution was analyzed and linked with the antifungal activity of the aromatic plant in PDA. The profile and the amount of phenolic compounds in the aqueous solution autoclaved for 20 min at 121 °C is shown in Table 2. It was observed that the highest number of phenolic compounds were present in the oregano aqueous solution, followed by Satureja and thyme. In general, aqueous solutions were rich in the phenolic compounds carvacrol and rosmarinic acid. The total amount of phenolic compounds in oregano, thyme and Satureja was 204.83, 121.99 and 156.82 µg/mL, respectively. This amount (if converted to dry matter) is higher compared to the respective amount reported in the literature for water-soluble phenolics from oregano, thyme and Satureja [19]. It appears that although some compounds (i.e., ferulic acid, sinapic acid, p-coumaric acid and trans-cinnamic acid) were negatively affected by sterilization at 121 °C, the extracted amount of other phenolic compounds was higher, suggesting a high sensitivity for these compounds to high temperature.

**Figure 1:**
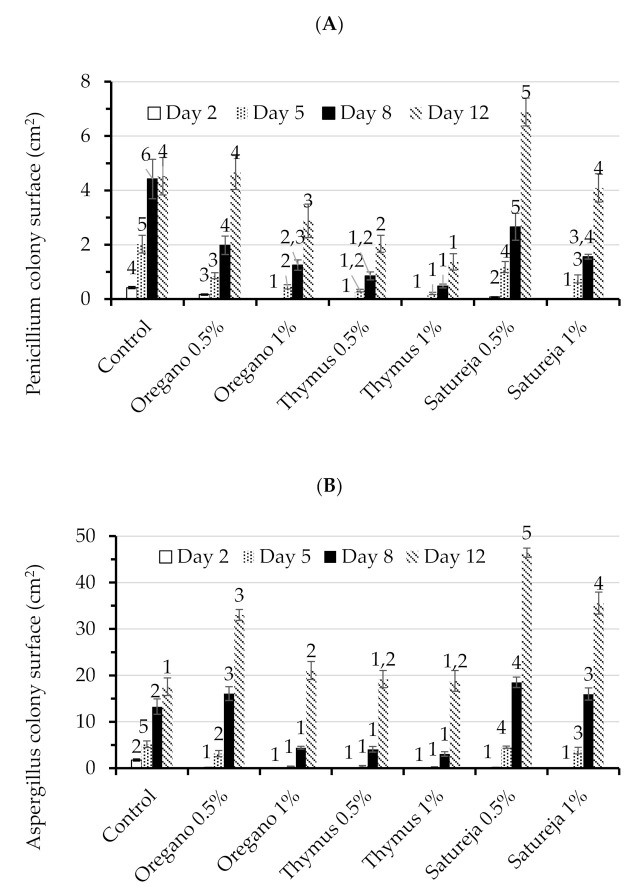
Average growth (cm^2^) in potato dextrose agar (PDA) medium for (**A**) *Penicillium* and (**B**) *Aspergillus* with treatment during 12 days of storage. Similar number superscripts (reported above each column) in the same incubation day are not significantly different (*p* < 0.05) by Duncan’s multiple range test.

The results of the antifungal activity of the studied aromatic plants in the dry form on PDA medium are shown in Figure 1. In general, the higher the content of aromatic plant, the higher the inhibition for each fungus studied. No fungal (*Aspergillus*, *Penicillium*) growth was observed after two days of storage at the highest concentration (1%) for all the aromatic plants studied. At the concentration of 0.5%, thyme efficiently inhibited 100% of the *Aspergillus* and *Penicillium* growth, while oregano and Satureja inhibited 91.92% and 92.10% of *Aspergillus* growth, and 60.94% and 81.54% of *Penicillium* growth, respectively.

After five days of storage, the maximum (95.03%) and minimum (12.92%) inhibition of *Aspergillus* was recorded for thyme 1% and Satureja 0.5%, respectively. After eight days of storage, two Satureja samples (at 0.5% and 1%) and oregano at 0.5% showed that the surface occupied by the *Aspergillus* colony was greater than that of the respective control (no added dry aromatic plants after eight days of storage). It seems that *Penicillium* growth was inhibited from a minimum of 39.82% (Satureja 0.5%) to a maximum of 89.15% (thyme 1%) in all PDA samples. After 12 days of storage, only PDA with thyme showed *Aspergillus* colony surfaces similar to that of the respective control, with the rest of the samples showing significantly higher values (Figure 1B). It seems that beyond this period, the addition of the aromatic plants promotes the growth of *Aspergillus*. Both PDA samples with thyme and the sample with oregano 1% inhibited the growth of *Penicillium* after 12 days of storage, whereas Satureja at 1% and oregano at 0.5% exhibited similar values to that of the control (Figure 1A). Only Satureja at 0.5% showed a colony surface area higher than the control.

Although a significant increase in the fungal surface with increasing storage was observed, thyme exhibited a higher inhibition effect, followed by oregano and Satureja. In general, the aromatic plants were significantly more efficient in inhibiting the *Penicillium* species than *Aspergillus*. It seems that at 0.5%, the dry aromatic plants encouraged *Aspergillus* growth since it was observed that the overall mean was significantly higher than that of the control. Contrary, the aromatic plants were effective in inhibiting the growth of *Penicillium* at the concentrations used.

### 3.3. Antifungal Activity in Bread

**Figure 2:**
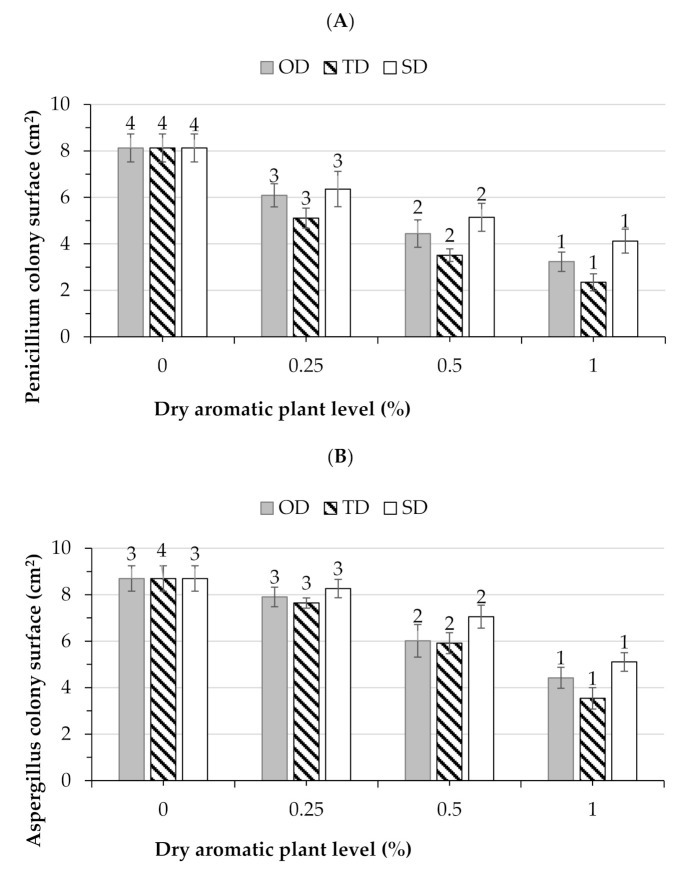
Average growth (cm^2^) in bread for (**A**) *Penicillium* and (**B**) *Aspergillus* following treatment with dried aromatic plants after 7 days of storage. OD—oregano, TD—thyme, and SD—Satureja dry matter. Similar number superscripts (reported above each column) for the same plant are not significantly different (*p* < 0.05) by Duncan’s multiple range test.

**Figure 3:**
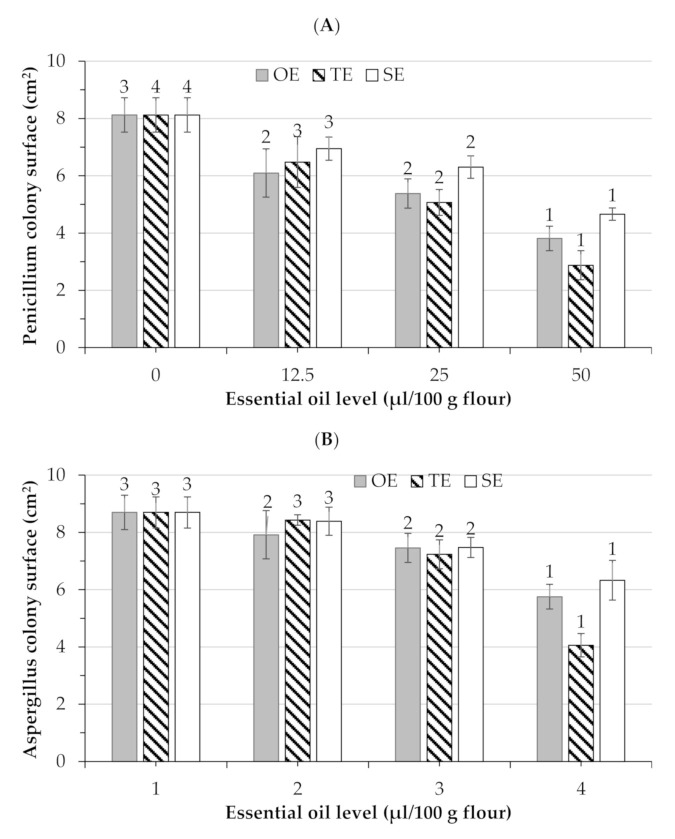
Average growth (cm^2^) in bread for (**A**) *Penicillium* and (**B**) *Aspergillus* following treatment with essential oils after 7 days of storage. OE—oregano, TE—thyme, and SE—Satureja essential oil. Similar number superscripts (reported above each column) in the same plant are not significantly different (*p* < 0.05) by Duncan’s multiple range test.

The antifungal properties of dry aromatic plants and their respective essential oils in bread are presented in Figure 2 and Figure 3. The addition of aromatic plants (dry matter as well as essential oil) in the bread recipe resulted in antifungal activity against both *Penicillium* and *Aspergillus*. Other recent studies have examined the use of essential oils in order to extend the shelf-life of bread. The authors of [32,33] used starch microcapsules containing essential oils (citral and eugenol) to inhibit *P. roqueforti* and *A. niger* and extended the shelf life of bread. On the other hand, Gonçalves da Rosa et al. [33] observed that the addition of essential oils from *Origanum vulgare Linneus* and *Thymus vulgaris* incorporated in nanocapsules also extended the shelf life of bread. Both studies demonstrated that the inclusion of essential oils in nanocapsules before their incorporation in the bread recipe represents a great potential for the preservation of baked foods. In the study by Vasileva et al. [34], the breads with 2.5% and 5% added lavender (*Lavandula angustifolia*) waste had increased shelf life compared to control bread without added plant waste, showing no fungal or bacterial spoilage for four days, indicating its potential as a bread preservative. They observed that there was no difference in antimicrobial/antifungal activity among the two levels of addition. In the present study, it was observed that the level of addition played a significant (*p* < 0.05) effect in decreasing the fungal colony surface, regardless of the type of fungus. The higher the level of addition, the higher the inhibition effect against the fungi. Among the aromatic plants, thyme had significant antifungal activity, with values of 53.34–71.15%, which was the greatest activity observed against *Penicillium* when added in the bread recipe in dry form and the least against *Aspergillus* when added as an essential oil. On the other hand, Satureja exhibited the lowest inhibition values against both fungi among the three aromatic plants. A general linear model applied to these data revealed that both forms of addition (essential oil and dry material) had the greatest activity (significance *p* < 0.5) against *Penicillium* compared to *Aspergillus*. In general, when the aromatic plants are added in the dry form in the bread recipe, they were more effective compared to essential oils.

The antimicrobial activity of the aqueous extract of aromatic plants may be attributed to the high content of phenolics. Among the compounds present in aromatic plants, carvacrol and thymol are recognized for their strong antifungal effect. They damage cell membranes by interacting with sterols, in particular with ergosterol [35]. This fact can explain the higher inhibition against fungi observed for thyme and oregano when added as essential oils compared to Satureja, but not the rate of inhibition (see levels of carvacrol and thymol in Table 1). The presence of compounds other than carvacrol and thymol, which were found in much lower levels, may have high inhibition capacity or they have synergistic/antagonistic effects, which could affect the inhibition capacity.

Plant phenolics, together with terpenoids, alkaloids, and lectins and polypeptides, are recognized to have antimicrobial properties [36]. Phenolic and flavonoid compounds possess both antifungal and antimicrobial activity [37,38,39]. Flavonoids have been reported to be involved in the inhibition of nucleic acid biosynthesis and other metabolic processes [40], as well as in the inhibition of the spore germination of plant pathogens [41]. Moreover, flavonoids are synthesized by plants in response to microbial infection. Phenolic compounds with a C3 side chain at a lower level of oxidation and containing no oxygen groups have often been reported to be antimicrobials [42]. Based on previously published data [19,20], it was expected that the inhibition of fungi should vary in the following range: oregano > thyme > Satureja, since total phenolics among the raw dry aromatic plants and in bread vary in this order (402.19, 379.49 and 357.24 μg/g bread, respectively). When added in dry form, theoretically, oregano should have shown the highest inhibition level, whereas Satureja should have shown the lowest. It seems that the profile of phenolic compounds, especially the amount of flavonoids that enriched the bread recipe, could affect the level of inhibition. Indeed, bread made with thyme (76.85 μg/g bread) showed the highest level of flavonoids compared to Satureja (72.99 μg/g bread) and oregano (41.05 μg/g bread) [20]. Thus, the final effect on the inhibition of fungi is a result of the combination of the profile of phenolic compounds with the level of each compound present.

## 4. Conclusions

Growth inhibitory potential against fungi by incorporating aromatic plants (in dry form or as essential oils) in food systems represent a very interesting approach that holds economic value. The enrichment of a bread recipe with aromatic plants, not only added as essential oils but also in dry form, could inhibit the mycelial growth of *Penicillium* and *Aspergillus* fungi at a concentration range where the breads are considered acceptable based on sensory tests. It was observed that aromatic plants were more efficient in the inhibition of the growth of *Penicillium* compared to *Aspergillus*. This presents an advantage since *Penicillium* represents the primary fungal contaminant of bread. Moreover, the addition of aromatic plants in the dry form in the bread recipe is more effective against both fungi studied compared to essential oils. However, further research is needed to evaluate the effect of bread recipe and processing parameters on the inhibition effect in order to further augment it. In conclusion, at the levels used in this stidy, aromatic plants in dry form or as essential oils may be useful as natural and safe additives for promoting the safety and quality of ready-to-eat bread.

## Figures and Tables

**Table 1:** Chemical composition (content %) of the essential oils of the three aromatic plants obtained by hydrodistillation and determined by GC/MS: *Origanum vulgare* ssp. *hirtum*, *Thymus capitatus* and *Satureja thymbra* *.

Compound	Elution Time (min)	*Origanum vulgare* ssp. *hirtum*	*Thymus capitatus*	*Satureja thymbra*
		Mean ± SD	Mean ± SD	Mean ± SD
Monoterpene hydrocarbons		12.62	19.35	44.99
α-thujene	7.8	0.64 ± 0.03 ^a^	0.69 ± 0.01 ^a^	1.45 ± 0.01 ^b^
α-pinene	8.1	0.78 ± 0.03 ^a^	0.84 ± 0.01 ^b^	1.17 ± 0.02 ^c^
camphene	8.8	0.15 ± 0.00 ^a^	0.16 ± 0.00 ^b^	0.31 ± 0.01 ^c^
β-pinene	10.1	0.14 ± 0.00 ^a^	0.14 ± 0.01 ^a^	0.40 ± 0.01 ^b^
cis-sabinene hydrate	16.0	- ± - ^a^	- ± - ^a^	0.09 ± 0.01 ^b^
trans-sabinene hydrate	18.1	- ± - ^a^	0.71 ± 0.04 ^b^	1.01 ± 0.05 ^c^
β-myrcene	10.9	1.61 ± 0.06 ^b^	1.97 ± 0.02 ^c^	0.33 ± 0.01 ^a^
α-phellandrene	11.8	0.19 ± 0.01 ^b^	0.29 ± 0.01 ^c^	0.09 ± 0.00 ^a^
β-phellandrene	13.3	0.38 ± 0.01 ^a^	0.60 ± 0.01 ^b^	0.67 ± 0.01 ^b^
α-terpinene	12.5	0.97 ± 0.04 ^a^	1.66 ± 0.04 ^b^	3.40 ± 0.01 ^c^
p-cymene	13.0	5.00 ± 0.07 ^a^	7.18 ± 0.16 ^c^	6.67 ± 0.01 ^b^
γ-terpinene	15.3	2.62 ± 0.05 ^a^	4.90 ± 0.11 ^b^	29.28 ± 0.19 ^c^
α-terpinolene	17.3	0.14 ± 0.01 ^b^	0.21 ± 0.00 ^c^	0.12 ± 0.00 ^a^
Oxygenated monoterpenes		1.12	1.17	0.98
borneol	23.6	0.25 ± 0.03 ^a^	0.28 ± 0.03 ^a^	0.38 ± 0.02 ^a^
terpinen-4-ol	24.6	0.87 ± 0.04 ^b^	0.89 ± 0.05 ^b^	0.60 ± 0.02 ^a^
Sesquiterpenes		2.14	1.94	4.92
trans-caryophyllene	44.4	1.23 ± 0.00 ^a^	1.80 ± 0.02 ^b^	4.52 ± 0.06 ^c^
α-humulene	46.4	- ± - ^a^	- ± - ^a^	0.19 ± 0.00 ^b^
β-bisabolene	49.8	0.91 ± 0.00 ^c^	0.14 ± 0.00 ^a^	0.21 ± 0.01 ^b^
Oxygenated sesquiterpenes				
caryophyllene oxide		- ± - ^a^	0.16 ± 0.01 ^b^	0.21 ± 0.01 ^c^
Monoterpene phenols		82.64	76.37	46.34
carvacrol	36	82.48 ± 0.62 ^c^	76.19 ± 0.18 ^b^	44.93 ± 0.35 ^a^
thymol	35.1	0.16 ± 0.03 ^a^	0.18 ± 0.01 ^a^	1.41 ± 0.02 ^b^
Alcohols				
1-octen-3-ol	10.3	0.45 ± 0.06 ^b^	0.42 ± 0.04 ^b^	0.21 ± 0.03 ^a^
Ketones				
3-octanone	10.8	0.15 ± 0.01 ^b^	- ± - ^a^	2.02 ± 0.04 ^c^
Total		99.10	99.37	97.18

* Values are means of duplicate measurements and reported with the respective standard deviation (SD); means with any similar superscripts in the same line are not significantly different (*p* = 0.05) by Duncan’s multiple range test.

**Table 2:** Content (µg/mL) of phenolic compounds in aqueous solutions containing 1% dry matter of three aromatic plants autoclaved for 20 min at 121 °C *.

Phytochemicals	*Origanum vulgare* ssp. *hirtum*	*Thymus capitatus*	*Satureja* *thymbra*
	Mean ± SD	Mean ± SD	Mean ± SD
Phenolic acids and their derivatives	90.53	22.66	55.44
4-hydroxybenzoic acid	0.23 ± 0.02 ^a^	0.32 ± 0.04 ^b^	1.02 ± 0.04 ^c^
gallic acid	0.14 ± 0.02 ^b^	0.36 ± 0.03 ^c^	0.08 ± 0.01 ^a^
protocatechuic acid	0.93 ± 0.06 ^b^	0.68 ± 0.04 ^a^	0.73 ± 0.05 ^a^
syringic acid	0.22 ± 0.02 ^a^	0.64 ± 0.02 ^c^	0.35 ± 0.04 ^b^
vanillic acid	0.75 ± 0.01 ^a^	1.05 ± 0.07 ^b^	0.75 ± 0.05 ^a^
trans-cinnamic acid	ND	ND	0.04 ± 0.01
caffeic acid	5.66 ± 0.09 ^b^	2.79 ± 0.06 ^a^	6.85 ± 0.03 ^c^
ferulic acid	ND	ND	ND
sinapic acid	ND	ND	ND
p-coumaric acid	ND	ND	ND
chlorogenic acid	0.27 ± 0.01 ^a^	0.34 ± 0.01 ^b^	ND
rosmarinic acid	82.34 ± 0.13 ^c^	16.48 ± 0.09 ^a^	45.61 ± 0.16 ^b^
Flavonoids	9.11	17.15	10.01
luteolin	0.41 ± 0.03	LLQ	LLQ
apigenin	ND	ND	ND
quercetin	ND	1.18 ± 0.07 ^b^	0.78 ± 0.04 ^a^
kaempferol	0.67 ± 0.02 ^a^	0.70 ± 0.04 ^a^	0.78 ± 0.03 ^b^
myricetin	ND	5.62 ± 0.09 ^b^	0.28 ± 0.05 ^a^
rutin	1.62 ± 0.01	ND	ND
(±)-naringenin	ND	ND	1.17 ± 0.03
(+)-catechin	ND	0.71 ± 0.03 ^a^	1.93 ± 0.12 ^b^
(−)-epicatechin	5.96 ± 0.08 ^b^	8.78 ± 0.18 ^c^	5.07 ± 0.14 ^a^
(−)-epigallocatechine	0.46 ± 0.05 ^b^	0.16 ± 0.03 ^a^	ND
Phenolic monoterpenes	105.19	82.17	91.37
carvacrol	105.19 ± 0.15 ^c^	82.17 ± 0.22 ^a^	91.37 ± 0.18 ^b^
thymol	ND	ND	ND
Total phenolics	204.83	121.99	156.82

ND—not detected; LLQ—lower than LOQ (Limit of quantification). * Values are means of duplicate analysis and reported with the respective standard deviation (SD); means with any similar superscripts in the same line are not significantly different (*p* = 0.05) by Duncan’s multiple range test.

## Abbreviations

(−)-epicatechin (EPI); (−)-epigallocatechine (EPIG); (+)-catechin (CAT); (±)-naringenin (NAR); 4-hydroxybenzoic acid (4-HBA); apigenin (API); caffeic acid (CA); carvacrol (CAR); chlorogenic acid (CLA); diode-array detector (DAD); ferulic acid (FA); gallic acid (GA); gas chromatography/mass spectrometry (GC/MS); generally recognized as safe (GRAS); high pressure liquid chromatography (HPLC); kaempferol (KAM); luteolin (LUT); myricetin (MYR); one-way analysis of variance (ANOVA); oregano dry matter (OD); oregano essential oil (OE); *Origanum vulgare* ssp. *hirtum* (oregano); p-coumaric acid (PCA); potato dextrose agar (PDA); protocatechuic acid (PRCA); quercetin (QUE); rosmarinic acid (RMA); rutin (RUT); Satureja dry matter (SD); Satureja essential oil (SE); *Satureja thymbra* (Satureja); sinapic acid (SA); syringic acid (SRA); thyme dry matter (TD); thyme essential oil (TE); thymol (THY); *Thymus capitatus* (thyme); trans-cinnamic acid (CIN); vanillic acid (VA).
